# Effect of Methanolic Leaf Extract of *Ocimum basilicum* L. on Benzene-Induced Hematotoxicity in Mice

**DOI:** 10.1155/2012/176385

**Published:** 2012-09-05

**Authors:** S. Saha, M. K. Mukhopadhyay, P. D. Ghosh, D. Nath

**Affiliations:** ^1^Cytogenetics & Plant Biotechnology Research Unit, Department of Botany, University of Kalyani, Nadia, Kalyani 741235, India; ^2^Cytogenetics & Molecular Biology Laboratory, Department of Zoology, University of Kalyani, Nadia, Kalyani 741235, India

## Abstract

The aim of the present study was to investigate the protective role of methanolic leaf extract of *Ocimum basilicum* L. against benzene-induced hematotoxicity in Swiss albino mice. GC analysis and subacute toxicity level of the extract were tested. Mice were randomly divided into three groups among which II and III were exposed to benzene vapour at a dose 300 ppm × 6 hr/day × 5 days/week for 2 weeks and group I was control. Group III of this experiment was treated with the leaf methanolic extract at a dose of 100 mg/kg body weight, a dose in nontoxic range. Hematological parameters (Hb%, RBC and WBC counts), cell cycle regulatory proteins expression and DNA fragmentation analysis of bone marrow cells was performed. There was an upregulation of p53 and p21 and downregulation of levels of CDK2, CDK4, CDK6, and cyclins D1 and E in leaf extract-treated group. DNA was less fragmented in group III compared to group II (P < 0.05). The present study indicates that the secondary metabolites of *O. basilicum* L. methanolic leaf extract, comprising essential oil monoterpene geraniol and its oxidized form citral as major constituents, have modulatory effect in cell cycle deregulation and hematological abnormalities induced by benzene in mice.

## 1. Introduction

Natural products have been investigated for promising new leads in pharmaceutical development [1, 2] as well as promising new source of therapeutic agents in plant secondary metabolites that characterize certain plants or plant group [3]. Interest in these secondary metabolites has been focused upon their medicinal properties [4]. Monoterpenes have long been recognized to possess significant medicinal values especially to cancer cells. Monoterpenes are nonnutritive dietary components found in the essential oils of citrus fruits, cherry, mint, and herbs. They function physiologically as chemoattractants or chemorepellents [5], and they are largely responsible for the distinctive fragrance of many plants. These 10 carbon isoprenoids are derived from the mevalonate pathway in plants.

A number of dietary monoterpenes have antitumor activity, exhibiting not only the ability to prevent the formation or progression of cancer, but also to regress existing malignant tumors. A variety of dietary monoterpenes have been shown to be effective in the chemoprevention and chemotherapy of cancer [6–10]. The cancer suppressing chemopreventive activity of monoterpenes during the promotion phase of mammary and liver carcinogenesis may be due to inhibition of tumor cell proliferation, acceleration of the rate of tumor cell death, and/or induction of tumor cell differentiation [11]. Now, monoterpene research is progressing into human clinical trials for chemotherapeutic activity from the last decade. Monoterpenes also possess many characteristics of ideal chemopreventive agents, namely, efficacious antitumor activity, commercial availability, low cost, oral bioavailability, and low toxicity, making it feasible to begin considering them for human cancer chemoprevention testing.

*Ocimum basilicum* L. popularly known as Tulsi in Hindi and “Sweet Basil” in English in one of the sacred herbs for Hindus in the Indian subcontinent. And India has one of the oldest, richest, and most diverse cultural living traditions associated with the use of medicinal plants [12]. The entire plant of *Ocimum basilicum *L. has medicinal value although mostly the leaves, and sometimes the seeds, are used. It has a versatile role in traditional medicine. Earlier studies in particular, the leaves of *Ocimum basilicum* L. offer promise to biologically active constituents such as insect repellent, nematocidal, antibacterial, antifungal and antioxidants properties [13–16]. It is also used as an antiemetic agent [17]. In addition, more recently [18], anticancer activity of *Ocimum basilicum* L. extract and its fractions was evaluated using human cancer cell lines.

 Benzene is an ubiquitous environmental chemical that cause different types of haematotoxicity. It is known to be a very volatile liquid, with several organic and inorganic constituents. These vapors constitute various components of pollutants in the air, which are of great environmental and human health concern. Exposures to these pollutants are common in the refineries, oil fields, refueling stations, petrochemical industries, and motor mechanical workshops. A good percent of human populace is directly or indirectly exposed to these pollutants in the course of their day-to-day activities. Generally, those occupationally exposed constitute the population at greater risk of frequent exposure [19, 20]. The potential health hazards associated with chronic or subchronic exposure to these ubiquitous pollutants in the environment has attracted the attention of the general public and scientific community in particular. 

Exposure of mice to benzene vapour induces inflammatory responses, oxidative stress, alterations in cell cycle progression and DNA damage, and so forth, which together have been implicated in various blood diseases, including the development of acute myeloid leukemia [21, 22]. In this regard, the benzene-exposed animal model can act as a significant template for the study of environmental-occupational cancers with respect to different parameters measuring clinical prevention intervention. The present study was designed to determine whether oral application of the methanolic leaf extracts *Ocimum basilicum* L. had any role in prevention of benzene-induced alterations in cell cycle progression in mouse bone marrow.

## 2. Materials and Methods

### 2.1. Plant Material Collection

Fresh leaves of *Ocimum basilicum *L. were collected from medicinal and aromatic plant garden, Department of Botany, University of Kalyani, Kalyani, India, in August of 2011, which is located at 22°57′N latitude, 88°22′E longitude with an average altitude of 9.75 m above mean sea level. The taxonomic identification of plant material was confirmed by Dr. G. G. Maity, Professor of Taxonomy, Taxonomy and Plant systematic Unit, Department of Botany, University of Kalyani. The voucher specimens (PDG/Bot. 136) was deposited and preserved in the Department of Botany, University of Kalyani, Kalyani, India, for reference.

### 2.2. Other Materials

The primary antibodies specific for cyclin D1, cyclin E, CDK2, CDK4, CDK6, Cip1/p21, p53 and *β*-actin and the secondary antibodies were purchased from Santa Cruz Biotechnology, Inc. (Santa Cruz, CA, USA). All other chemicals used in our experiments were of analytical grade. Blood collected from subacute treatment group was used for the estimation of serum biochemical parameters like serum glutamate oxaloacetate transaminase (SGOT), serum glutamate pyruvate transaminase (SGPT), serum alkaline phosphatase (ALP), serum total cholesterol, total protein, urea, uric acid, and creatinine contents by using reagent kits (Span Diagnostics, Surat, India), following the instructions of manufacturer.

### 2.3. Experimental Animals

Six- to eight-week-old Swiss albino male mice (*Mus musculus*) were purchased from a reputed supplier at a regular basis from maintained strain and were housed in a stainless steel wire cages (Tarsons, India) and maintained on a 12-hour light-dark cycle. Pellet diet (West Bengal Diary and Poultry Development Corp. Ltd., Kalyani Industrial Area, Kalyani) was provided *ad libitum *except during the 6-hour daily period of benzene inhalation. Water was supplied *ad libitum* automatically through the tubing throughout the study. 

Benzene (MERCK, India) vapour was generated by heating liquid benzene to 16°C and channeled into the inhalation chamber. The groups of experimental mice were exposed 300 ppm, benzene for 6 hr/day, 5 days/week for 2 weeks in 1.3 m^3^ inhalation chambers (S.B. Equipments, WB, India) and one control group was exposed to ambient air for the same time duration, interval, and periods. Benzene concentration in the chambers was monitored at half-hour. intervals during daily exposures. The temperature and humidity in the chambers were automatically maintained at 24 ± 1°C and 55 ± 10%, respectively. This animal can act as a model for benzene-exposed haematotoxicity and leukemogenesis leading to secondary acute myelocytic leukemia (unpublished data).

### 2.4. Preparation of Plant Extract

Secondary metabolites, including flavonoids and other phenolic compounds, were extracted from dry leaves of *Ocimum basilicum *L. Collected plant material was dried in shade and ground in a grinder with a 2 mm diameter mesh. The dried and powdered plant materials (100 gm) were extracted successively using with 500 ml of methanol (1 : 5 w/v) by using Soxhlet extractor for 24 hours at a temperature not exceeding the boiling point of the solvent [23]. The extracts were filtered using Whatman filter paper (Number 1) and then concentrated in vacuo at 40°C using a Rotary evaporator. The residues obtained were stored in dark glass bottles at −20°C in air tight containers for further tests. The yield of the oils was calculated based on dried weight of plant materials. 

### 2.5. GC-MS and GC-FID Analysis

GC-MS analyses of the essential oils were performed on a Hewlett-Packard gas chromatograph, model 6890, equipped with a FID, using HP-5MS capillary column (30 m length × 0.25 mm i.d × 0.25 *μ*m film thickness), Agilent Technologies, USA. The injector was set at 250°C and the detector at 280°C, respectively. Oven temperature of the chromatogram was raised from 60°C to 280°C, respectively, at the heating rate of 10°C/min then isothermally held for 5 min. The injector volume was 1.0 *μ*L. The solvent delay was 2 min and injected in a split ratio of 1 : 10. Helium was used as carrier at 15 P.S.I. inlet pressure. The essential oil constituents were identified by comparison of their GC retention indices (RI) and mass spectra with those of the authentic standard compounds. Quantification of the relative amount of the individual components was performed according to the area percentage method. 

### 2.6. Subacute Toxicity Study of the Plant Extract

The crude methanolic leaf extracts were evaluated for their toxicity in *O. basilicum *L. noninfected male Swiss albino mice aged six to eight weeks and weighing 22–28 g. For the test of toxicity of the extract, 30 mice were randomly divided into three groups of ten animals (5 males and 5 females) per cage. Before oral administration of a dose, the mice were fasted for one to two hrs [24]. Then the extract was dissolved in distilled water and administered daily by oral gavage to the mice, groups II and III and the control group (group I) receiving respective volume of distilled water (vehicle), for 29 days. The mice in group II were given orally the extract of *O. basilicum *L. at a dose of 50 mg/kg, and the mice in group III were given the extract at dose of 1500 mg/kg of body weight. Then, the mice were observed continuously for one hr after the treatment; intermittently for four hrs, and thereafter over a period of 29 days. The mice were observed for gross behavioral changes, coat conditions, discharge, movement, body weight changes, serum biochemical parameters, hematological parameters, and mortality for 30 days to test subacute toxicity.

### 2.7. Hematological Parameters

After completion of the treatment with plant extract, the animals were made to fast overnight and sacrificed on the 30th day by decapitation and blood was collected. Blood hemoglobin concentration (g%) was determined using Sahli's hemoglobinometer and total WBC and RBC were counted using Neubauer's hemocytometer [25]. Other blood parameters including platelet count, differential count of monocyte, lymphocyte, neutrophil and differentials of RBC such as hematocrit, MCV, MCH, and MCHC were performed.

### 2.8. Bone Marrow Sampling

The femur bone of the sacrificed experimental mice was dissected and both the proximal and distal ends were removed. Phosphate buffer solution of pH 7.2 was injected gently into one end of the shaft. This process was followed to flush out the bone marrow through the opposite end into a collection vessel to prepare single cell suspension within one hour of stopping benzene inhalation. 

### 2.9. Immunoblotting

Protein extracts of the bone marrow were prepared using 5 different animals individually by sonicating femoral bone marrow cells in a cell lysis buffer containing 20% SDS, 2 mM phenylmethyl sulphonyl fluoride, and a protease inhibitor/phosphate inhibitor [26]. Protein concentration was quantified using Bradford method [27]. Protein extracts from bone marrow (15 *μ*g for p21 and 10 *μ*g for other) were denatured, subjected to 12% (W/V) SDS-PAGE, and then transferred to PVDF membranes. After blocking nonspecific binding sites by incubating the membranes with 5% nonfat dried milk and 0.1% Tween 20 in Tris buffered saline (TTBS, pH 7.4) for 1 hour at room temperature, the membranes were incubated overnight at 4°C in the presence of diluted primary antibodies. The membranes were then washed with TTBS and incubated with 1 : 2000 horseradish-peroxidase conjugated secondary antibodies for 50 minutes at room temperature. To visualize the bands, the membranes were treated with a detection reagent for 1 minute. Band densities were measured using an image analyzer (image J, NIH).

### 2.10. DNA Fragmentation Assay

DNA was extracted from bone marrow single cell preparation (as mentioned before) using reagents and protocol suggested by Bangalore Genei (PI.NO: KT23). DNA concentration was measured at 260 nm using a spectrophotometer (Varian, Germany). DNA extracts were then subjected to 0.8% agarose gel electrophoresis using 1X TAE buffer as running buffer (composition: 40 mM Tris-acetate, 1mM EDTA) and ethidium bromide (final concentration into gel 0.5 *μ*g/mL) as staining agent. 5 *μ*L of 100 bp DNA ladder was loaded into one well in each assay. Finally, the gel was visualized and photographed using a gel documentation system (Genei image system).

### 2.11. Treatment of Benzene-Exposed Mice with Crude Methanolic Leaf Extract

Since no sign of toxicity of the plant extract was found in both low- and high-dose of plant extract-treated group, benzene-exposed mice were treated with the extract at a dose of 100 mg/kg body weight orally from the date of commencement of benzene exposure for 4 weeks daily. Control group of mice were treated with respective volume of distilled water containing 10% DMSO following the same schedule. During treatment, mice were supplied pelleted feed and water *ad libitum* (Table 1).

### 2.12. Ethical Clearance

The protocol used in this study was approved by the animal ethical committee, Department of Zoology, University of Kalyani (under Committee for the Purpose of Control and Supervision of Experiments on Animals, India). 

### 2.13. Statistical Analysis

The two-tailed Students *t*-test was done to determine significance of differences between benzene-exposed and plant leaf extract-treated group to control group. Differences considered as significance at *P* < 0.05 level.

## 3. Results and Discussion

### 3.1. Essential Oil Profiles of *O. basilicum* L. Leaves

**Figure 1:**
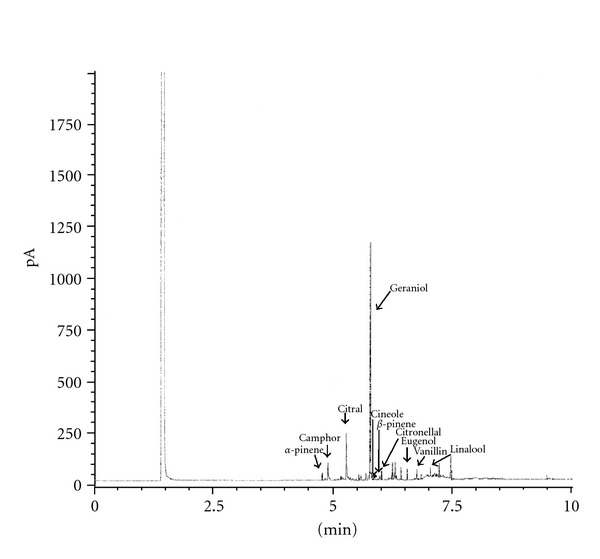
Chromatograms GC analysis of essential oils from *Ocimum basilicum *L.

The simple quantitative analysis of the crude methanolic extract showed remarkable variations in essential oil composition of *O. basilicum* L. from West Bengal. The yield of the essential oils obtained from dry leaf of *O. basilicum* L. was 1.77% (w/v) and the dominant or major constituents in sample were geraniol and citral ranging between 34.89% and 23.51% of the total oils (Table 2, Figure 1). Although methyl chavicol, linalool, methyl cinnamate, methyleugenol, eugenol, and geraniol are reported as major components of the oils of different chemotypes of *O. basilicum* L. [28–30]. The variation in the oil composition could be attributed to differences in soil conditions and altitude. It was observed that linalool and geraniol are the main components in *Ocimum basilicum* L. from Bangladesh, and the authors concluded that the oil composition could be dependent on climatic conditions [31]. In the present study, our results showed that geraniol and citral and the main constituents of *O. basilicum* L. in respect of West Bengal are condition. The observed variations may be due to different environmental and genetic factors and the nutritional status of the plants as well as other factors that can influence the oil composition.

### 3.2. Evaluation of Nontoxicity of Plant Extract

 Physicochemical observations were recorded to determine potential toxic effects of infused plant material in the animals. No adverse effect such as behavioral changes, coat conditions alterations, discharge and movement abnormality was seen for both group II and group III animals. No mice were found to be died during the study and nonsignificant changes of body weight, serum biochemical parameters, and hematological parameters (Table 3) were due to normal growth of the animals. So, the selected dose of plant extract is nontoxic.

### 3.3. Plant Material Arrest Benzene-Induced Cell Cycle Deregulation

**Figure 2:**
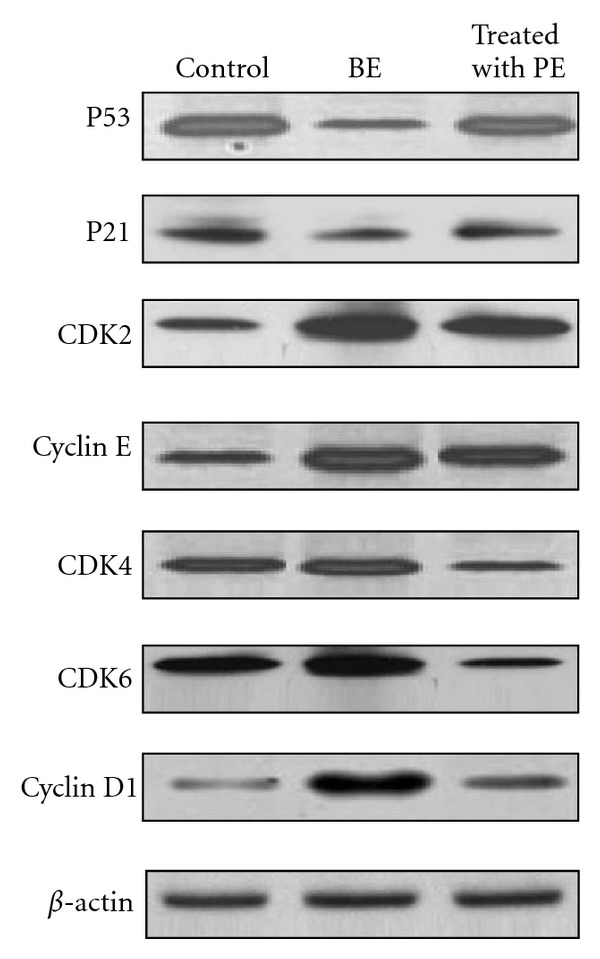
Immunoblotting of p53, p21, CDK2, cyclin E, CDK4, CDK6, cyclin D1. Panel 1: proteins from control animal; panel 2: proteins from benzene exposed animal; panel 3: proteins from animal treated with plant extract.

Reduced expression of cell cycle regulatory proteins such as p53 and p21 has been implicated in benzene-induced hematotoxicity in mice [26]. Based on our results, we hypothesized that the plant extract might have modulatory effects on cell cycle regulatory proteins. As shown in Figure 2, it is evident that the expression levels of cyclins (cyclins D1, E), CDK2, CDK4, and CDK6 were considerably higher in benzene-induced mice compared with control mice. However, treatment of mice with the material after benzene exposure resulted in inhibition of benzene-induced expression levels of cyclins (cyclins D1, E), CDK2, CDK4 and CDK6 compared with proteins obtained from nontreated mice. The level of Cip1/p21, which is a universal inhibitor of cell cycle progression, is transcriptionally activated by p53 after benzene exposure [26, 32]. Treatment of plant material resulted in the enhancement of the levels of p21 and p53 in benzene-exposed mice compared with nontreated benzene exposed mice. 

Mice exposed to benzene affect cell cycle regulators in bone marrow and blood. Regulation of cyclin-CDK complexes plays a key role in cell cycle progression at different phases in which CDKs are negatively regulated by a group of functionally related proteins known as CDK inhibitors, such as Kip/Cip family members [33–35]. p21 is a universal CDK inhibitor and p21 inhibits DNA replication process [36, 37], whereas p53 is upregulated in response to antiproliferative signals. Consistent with these reports, methanolic leaf extract treatment resulted in an upregulation of p53 and p21 and significantly reduced level of CDK4 and CDK6, cyclin D1, and moderately reduced level of CDK2 and cyclin E in benzene-induced animal with hematotoxicity, which may have caused cell cycle arrest mainly in G2 phase in hematopoietic cells. Thus, it can be suggested that modulation in cell cycle progression and inhibition of cell proliferation could be one of the possible mechanisms through which the leaf extract inhibits benzene-induced cell cycle deregulation.

### 3.4. Hematological Parameters

The results shown in Table 4 indicate that the number of RBC, WBC, and Hb (g%) has been decreased in benzene-exposed group compared to control group of animals. Hb (g%), RBC and WBC have been decreased to 9.96 ± 1.3, 2.11 ± 0.19, and 3.28 ± 0.45, respectively. After treatment of benzene-exposed mice with the plant leaf extract the above parameters increase significantly (*P* < 0.05) and the value reaches to 11.96 ± 1.22, 3.26 ± 0.33, and 4.67 ± 0.31, respectively. This result is also in accordance with overexpression of p21 in plant extract-treated group of animals as shown in immunoblot analysis. Among total WBC count, lymphocyte count increases significantly (*P* < 0.05) after treatment with the plant extract. Hct as RBC differential also increased significantly (*P* < 0.05) after treatment (Table 5).

Blood parameters analysis is relevant to risk evaluation as the hematological system has a higher predictive value for toxicity in humans (91%) when assay involve rodents and nonrodents [38]. Blood is an important index of physiological and pathological status in man and animals and the parameters usually measured are hemoglobin, total red blood cell (RBC), and leukocyte (WBC) counts [39]. The hematotoxicity of benzene is characterized by suppression of erythromyelopoiesis, resulting in the depression of leukocyte (lymphocyte count mainly) and erythrocyte levels in peripheral blood [32, 40]. This result is also in accordance with overexpression of p21 as shown in immunoblot analysis (Figure 2). But after treatment with the plant material, decrease in blood parameters was found to be less.

### 3.5. DNA Fragmentation Analysis

**Figure 3:**
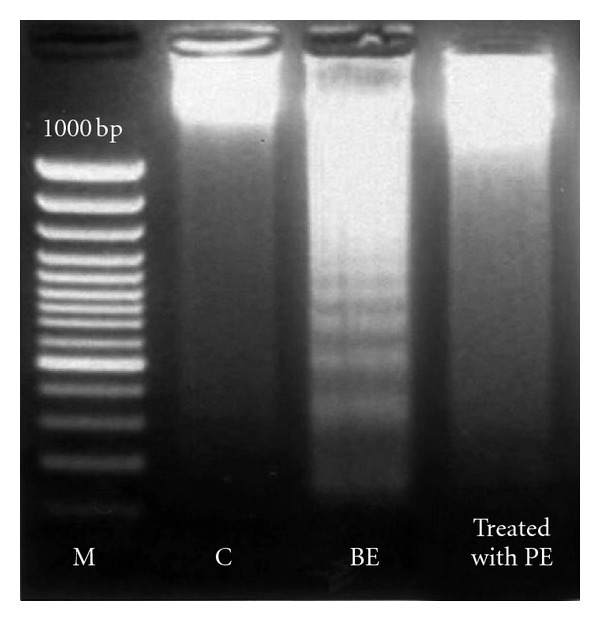
DNA fragmentation analysis (lane 1, M: marker; lane 2, control DNA; lane 3: DNA from benzene-exposed group; lane 4: DNA from plant extract-treated group).

As shown in Figure 3, DNA fragmentation assay of bone marrow cells characteristic of apoptosis was clearly detected after benzene exposure to mice and it is being less fragmented in methanolic leaf extract treated group. The data were coincident with the results of the cell cycle regulating kinases.

## 4. Conclusion

In conclusion, protection by plant secondary metabolites is currently an important strategy for controlling the process of various diseases including hematotoxicity. Therefore, there is a need to explore medicinal plants or other natural agents that can work as protective agents against hematotoxicity. In the present work, our results show significant activity of methanolic leaf extract of *Ocimum basilicum* L. against benzene-induced hematotoxicity in mice. These extract may promote chemopreventive activity in human. On the basis of above evidence, it is possible that monoterpenes like geraniol, citral, and eugenol, present in the extract may be responsible for this activity. However, this claim demands extensive studies on synergistic pharmacodynamic interactions of the phytochemicals as well as the effects on signal modulation which are essential for the development of multiactive natural drugs from *Ocimum basilicum *L. for cancer chemoprevention in the near future.

## Figures and Tables

**Table 1:** Treatment schedule.

Groups	Treatment
I	Not received any treatment
II	Benzene exposed, received only vehicle, distilled water containing 10% DMSO
III	Benzene exposed, received plant extract suspended in distilled water containing 10% DMSO at a dose of 100 mg/kg body weight for 4 weeks

**Table 2:** Percentage composition of the essential oils of *Ocimum basilicum *L. cultivated in West Bengal.

Compound	^ a^Retention indices (RI) in min.	^ b^Essential oil (%)
*α*-Pinene	4.76	0.23
Camphor	4.87	0.64
Citral	5.26	23.51
Geraniol	5.76	34.89
Cineole	5.83	0.05
*β*-Pinene	5.92	0.19
Citronellal	5.96	0.59
Eugenol	6.57	1.33
Vanillin	6.72	0.27
Linalool	7.41	2.21
Methanol	—	—

^a^Identification of oil components was based on their relative retention indices (retention times) with those of authentic standards.

^b^Quantitative estimation was done by analysis of FID area percent data.

**Table 3:** Evaluation of nontoxicity of the plant material. Effect of leaf methanolic extract on serum biochemical, hematological parameters, and body weight after subacute administration. Data are represented as mean ± SEM.

Parameters	Control	Lower dose (50 mg/Kg body weight)	Higher dose (1500 mg/Kg body weight)
	(group I)	(group II)	(group III)
SGOT (IU/dL)	40.75 ± 1.12	41.08 ± 1.19	40.96 ± 1.15
SGPT (IU/ dL)	34.22 ± 1.19	34.98 ± 1.16	35.11 ± 1.13
ALP (IU/dL)	81.12 ± 1.78	80.56 ± 1.76	80.01 ± 1.72
Bilirubin (mg/dL)	0.86 ± 0.13	0.85 ± 0.11	0.86 ± 0.12
Cholesterol (mg/dL)	149.25 ± 9.3	150.33 ± 8.7	150.69 ± 8.9
Total protein (mg/dL)	6.90 ± 2.09	6.89 ± 2.01	6.98 ± 2.33
Urea (mg/dL)	41.64 ± 1.32	42.09 ± 1.21	42.67 ± 1.31
Uric acid (mg/dL)	5.48 ± 1.77	5.12 ± 1.56	5.19 ± 1.67
Creatinine (mg/mL)	0.93 ± 0.12	0.91 ± 0.21	0.92 ± 0.18
RBC (× 10^12^/L)	6.6 ± 0.12	6.6 ± 0.16	6.7 ± 0.13
Hb (g/dL)	13.2 ± 0.22	13.1 ± 0.28	12.9 ± 0.31
WBC (× 10^9^/L)	31.3 ± 0.42	31.2 ± 0.0.38	32.1 ± 0.26
Platelet (× 10^9^/L)	756 ± 41.2	736 ± 46.1	750 ± 42.4
Body weight (g)	23 ± 1.2	23 ± 1.5	23 ± 1.4

**Table 4:** Variation of blood parameters among BE (benzene exposed) mice and treated with PE (plant extract) group. Data are expressed as mean ± SEM.

Group	Hb (g%)	RBC (× 10^12^/L)	WBC (× 10^9^/L)
Control	13.29 ± 2.1	4.08 ± 0.38	8.21 ± 1.2
BE	9.96 ± 1.3	2. 11 ± 0.19	3.28 ± 0.45
Treated with PE	11.96 ± 1.22*	3.26 ± 0.33*	4.67 ± 0.31*

*Significant increase (*P* < 0.05) in parameters.

**Table 5:** Variation of differential blood parameters among BE (benzene-exposed) mice and treated with PE (plant extract) group. Data are expressed as mean ± SEM.

Group	Differential leukocyte count			Differential of RBC				Platelet count
	Neutrophil (%)	Monocyte (%)	Lymphocyte (%)	Hct (%)	MCV (*μ*m^3^)	MCH (pg)	MCHC (g/dL)	(× 10^9^/L)
Control	22.03 ± 1.98	1.92 ± 0.06	68.34 ± 5.12	38.09 ± 2.11	52.78 ± 3.46	16.22 ± 0.12	26.44 ± 0.35	750 ± 38.21
BE	21.68 ± 1.32	1.88 ± 0.05	48.49 ± 3.51	10.23 ± 0.96	47.55 ± 2.98	14.11 ± 0.14	24.33 ± 0.38	798 ± 40.08
Treated with PE	21.92 ± 1.41	1.90 ± 0.06	62.12 ± 4.98*	24.35 ± 1.67*	48.34 ± 2.76	14.56 ± 0.13	24.49 ± 0.36	776 ± 39.97

*Significant increase (*P *<* *0.05) in parameters.
